# Dicyclo­hexyl­ammonium 3-[(hy­droxy­meth­yl)carbamo­yl]propano­ate

**DOI:** 10.1107/S1600536810044958

**Published:** 2010-11-06

**Authors:** Subadra Rajeswari, Ganesan Venkatesa Prabhu, Dhanapal Tamilvendan, Helen Stoeckli-Evans

**Affiliations:** a Department of Chemistry, National Institute of Technology, Tiruchirappalli 620 015, Tamil Nadu, India; b Institute of Physics, University of Neuchâtel, rue Emile-Argand 11, CH-2000 Neuchâtel, Switzerland

## Abstract

The title compound, C_12_H_24_N^+^·C_5_H_8_NO_4_^−^, contains one dicyclo­hexyl­ammonium cation and one 3-[(hy­droxy­meth­yl)carbamo­yl]propano­ate anion in the asymmetric unit. In the crystal, the ions are linked by inter­molecular N—H⋯O and O—H⋯O hydrogen bonds, forming chains propagating along [100].

## Related literature

For the biological activity of succinimide derivatives, see: Argay *et al.* (1999 ▶). For the preparation of the Mannich base 1-[(dicyclo­hexyl­amino)­meth­yl]pyrrolidine-2,5-dione, see: Tra­montini (1973 ▶); Tramontini & Angliolini (1990 ▶). For standard bond lengths, see: Allen *et al.* (1987 ▶).
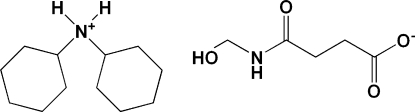


## Experimental

### 

#### Crystal data

C_12_H_24_N^+^·C_5_H_8_NO_4_^−^
*M* *_r_* = 328.45
Monoclinic, 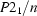

*a* = 5.6844 (5) Å
*b* = 17.7967 (12) Å
*c* = 18.4264 (16) Å
β = 95.495 (7)°
*V* = 1855.5 (3) Å^3^
*Z* = 4
Mo *K*α radiation
μ = 0.08 mm^−1^
*T* = 173 K
0.45 × 0.45 × 0.13 mm

#### Data collection

Stoe IPDS-2 diffractometer
Absorption correction: multi-scan (*MULscanABS* in *PLATON*; Spek, 2009 ▶) *T*_min_ = 0.714, *T*_max_ = 1.000
11855 measured reflections
3941 independent reflections
2568 reflections with *I* > 2σ(*I*)
*R*_int_ = 0.055

#### Refinement

*R*[*F* ^2^ > 2σ(*F* ^2^)] = 0.041
*wR*(*F* ^2^) = 0.095
*S* = 0.93
3941 reflections
225 parameters
H atoms treated by a mixture of independent and constrained refinement
Δρ_max_ = 0.19 e Å^−3^
Δρ_min_ = −0.16 e Å^−3^

### 

Data collection: *X-AREA* (Stoe & Cie, 2009 ▶); cell refinement: *X-AREA*; data reduction: *X-RED32* (Stoe & Cie, 2009 ▶); program(s) used to solve structure: *SHELXS97* (Sheldrick, 2008 ▶); program(s) used to refine structure: *SHELXL97* (Sheldrick, 2008 ▶); molecular graphics: *ORTEP-3* (Farrugia, 1997 ▶) and *Mercury* (Macrae *et al.*, 2006 ▶); software used to prepare material for publication: *SHELXL97*, *PLATON* (Spek, 2009 ▶) and *publCIF* (Westrip, 2010 ▶).

## Supplementary Material

Crystal structure: contains datablocks global, I. DOI: 10.1107/S1600536810044958/fj2363sup1.cif

Structure factors: contains datablocks I. DOI: 10.1107/S1600536810044958/fj2363Isup2.hkl

Additional supplementary materials:  crystallographic information; 3D view; checkCIF report

## Figures and Tables

**Table 1:** Hydrogen-bond geometry (Å, °)

*D*—H⋯*A*	*D*—H	H⋯*A*	*D*⋯*A*	*D*—H⋯*A*
N1—H1*A*⋯O1^i^	0.892 (17)	2.597 (17)	3.285 (2)	134.7 (14)
N1—H1*A*⋯O2^i^	0.892 (17)	1.975 (17)	2.8546 (18)	168.7 (15)
N1—H1*B*⋯O1^ii^	0.95 (2)	1.80 (2)	2.740 (2)	174.3 (17)
N2—H2*N*⋯O2^iii^	0.829 (17)	2.069 (17)	2.8914 (18)	171.1 (16)
O4—H4*O*⋯O3^iii^	0.88 (2)	1.78 (2)	2.6423 (16)	166.4 (19)

Symmetry codes: (i) 
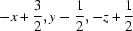
; (ii) 
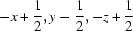
; (iii) 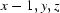.

**Fig. 1.**
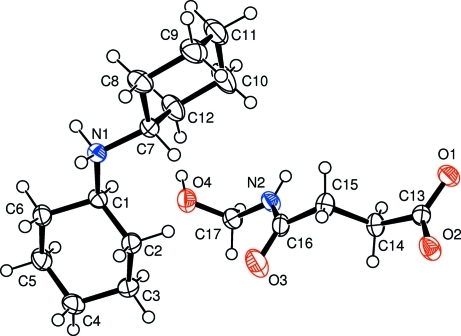
The molecular structure of the title compound, with displacement ellipsoids drawn at the 50% probability level.

**Fig. 2.**
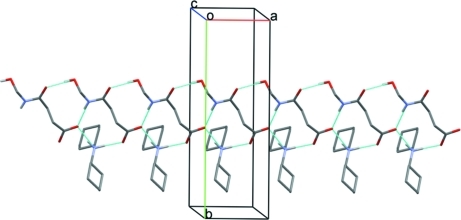
A partial view of the crystal packing of the title compound, showing the formation of the N—H···O and O—H···O hydrogen bonded (dashed cyan lines) polymer chain propagating in [100]; see Table 1 for details. H-atoms not involved in hydrogen bonding have been omitted for clarity.

## supplementary crystallographic information

### Comment

The pyrrolidine skeleton occurs in many families of biologically important compounds, and several succinimide derivatives are important in biology due to their antepileptic, anticonvulsive, fungicidal and other pharmalogical properties (Argay *et al.* 1999). The title compound was obtained during our attempts to prepare the Mannich base 1-((dicyclohexylamino)methyl)pyrrolidine-2,5-dione according to the reported procedure (Tramontini, 1973; Tramontini & Angliolini, 1990). The anion is probably formed by the hydrolysis of succinimide to yield the amino acid, *i.e.* NH_2_COCH_2_CH_2_COOH. The formation of the the title compound can be accounted for by the reaction of this amino acid with formaldehyde and the subsequent protonation of dicyclohexylamine.

The molecular structure of the title compound is illustrated in Fig. 1. It is compossed of a dicyclohexylammonium cation and a 4-(hydroxymethylamino)-4-oxobutanoate anion. The bond lengths (Allen *et al.*, 1987) and angles are normal.

In the crystal the cations and anions are linked *via* N—H···O and O—H···O hydrogen bonds involving both the cation and the anion. In this manner hydrogen bonded polymer chains are formed propagating in [100]; see Fig. 2 and Table 1 for details.

### Experimental

Dicyclohexylamine (36.2 ml, 0.2*M*) was added slowly to a solution of succinimide in ethanol (19.8 g, 0.2*M*). A solution of formaldehyde (40%, 15 ml) was added in drops with continuous stirring of the solution. The yellowish brown compound formed was initially sticky in nature and slowly turned into a stony mass, which was then crushed to form a fine powder. This product was washed several times with acetone and was then dried in the air in an oven at 333 K and recrystallized using water.

### Refinement

The H-atoms could all be located in difference electron-density maps. The NH_2_, NH and OH H-atoms were freely refined. O—H = 0.88 (2) Å, N—H = 0.829 (17) - 0.95 (2) Å. The C-bound H-atoms were included in calculated positions and treated as riding: C—H = 0.99 and 1.0 Å for CH_2_ and CH H-atoms, respectively, with *U*_iso_(H) = 1.2Ueq (parent C-atom).

### Crystal data

**Table:**

C_12_H_24_N^+^·C_5_H_8_NO_4_^−^	*F*(000) = 720
*M**_r_* = 328.45	*D*_x_ = 1.176 Mg m^−^^3^
Monoclinic, *P*2_1_/*n*	Mo *K*α radiation, λ = 0.71073 Å
Hall symbol: -P 2yn	Cell parameters from 7150 reflections
*a* = 5.6844 (5) Å	θ = 1.6–27.2°
*b* = 17.7967 (12) Å	µ = 0.08 mm^−^^1^
*c* = 18.4264 (16) Å	*T* = 173 K
β = 95.495 (7)°	Plate, colourless
*V* = 1855.5 (3) Å^3^	0.45 × 0.45 × 0.13 mm
*Z* = 4	

### Data collection

**Table:**

Stoe IPDS-2 diffractometer	3941 independent reflections
Radiation source: fine-focus sealed tube	2568 reflections with *I* > 2σ(*I*)
graphite	*R*_int_ = 0.055
φ and ω scans	θ_max_ = 26.7°, θ_min_ = 1.6°
Absorption correction: multi-scan (MULscanABS in *PLATON*; Spek, 2009)	*h* = −6→7
*T*_min_ = 0.714, *T*_max_ = 1.000	*k* = −22→21
11855 measured reflections	*l* = −23→23

### Refinement

**Table:**

Refinement on *F*^2^	Secondary atom site location: difference Fourier map
Least-squares matrix: full	Hydrogen site location: inferred from neighbouring sites
*R*[*F*^2^ > 2σ(*F*^2^)] = 0.041	H atoms treated by a mixture of independent and constrained refinement
*wR*(*F*^2^) = 0.095	*w* = 1/[σ^2^(*F*_o_^2^) + (0.0463*P*)^2^] where *P* = (*F*_o_^2^ + 2*F*_c_^2^)/3
*S* = 0.93	(Δ/σ)_max_ < 0.001
3941 reflections	Δρ_max_ = 0.19 e Å^−^^3^
225 parameters	Δρ_min_ = −0.16 e Å^−^^3^
0 restraints	Extinction correction: *SHELXL97* (Sheldrick, 2008), Fc^*^=kFc[1+0.001xFc^2^λ^3^/sin(2θ)]^-1/4^
Primary atom site location: structure-invariant direct methods	Extinction coefficient: 0.0046 (12)

### Special details

**Table:**

Geometry. Bond distances, angles *etc*. have been calculated using the rounded fractional coordinates. All su's are estimated from the variances of the (full) variance-covariance matrix. The cell e.s.d.'s are taken into account in the estimation of distances, angles and torsion angles
Refinement. The H-atoms could all be located in difference electron-density maps. The NH_2_, NH and OH H-atoms were freely refined. O—H = 0.88 (2) Å, N—H = 0.829 (17) - 0.95 (2) Å. The C-bound H-atoms were included in calculated positions and treated as riding: C—H = 0.99 and 1.0 Å for CH_2_ and CH H-atoms, respectively, with *U*_iso_(H) = 1.2Ueq (parent C-atom).

### Fractional atomic coordinates and isotropic or equivalent isotropic displacement parameters (Å^2^)

**Table:**

	*x*	*y*	*z*	*U*_iso_*/*U*_eq_	
N1	0.1835 (3)	0.16377 (7)	0.26459 (6)	0.0231 (4)	
C1	0.1626 (3)	0.15553 (8)	0.18284 (7)	0.0240 (4)	
C2	0.3908 (3)	0.17650 (9)	0.15118 (7)	0.0302 (5)	
C3	0.3685 (3)	0.16325 (9)	0.06882 (8)	0.0337 (5)	
C4	0.2964 (3)	0.08243 (9)	0.05011 (8)	0.0376 (5)	
C5	0.0679 (3)	0.06210 (9)	0.08250 (8)	0.0360 (5)	
C6	0.0916 (3)	0.07455 (8)	0.16494 (7)	0.0295 (5)	
C7	0.2441 (3)	0.24038 (8)	0.29523 (7)	0.0268 (5)	
C8	0.2700 (3)	0.23401 (9)	0.37808 (8)	0.0342 (5)	
C9	0.3228 (3)	0.31025 (10)	0.41315 (9)	0.0418 (6)	
C10	0.1112 (5)	0.37352 (10)	0.30517 (10)	0.0554 (7)	
C11	0.1319 (4)	0.36647 (10)	0.38784 (9)	0.0461 (6)	
C12	0.0575 (4)	0.29729 (9)	0.26847 (8)	0.0402 (6)	
O1	0.7522 (2)	0.63034 (6)	0.19198 (6)	0.0350 (3)	
O2	1.02645 (19)	0.55189 (6)	0.16058 (6)	0.0358 (3)	
O3	0.5058 (2)	0.35217 (7)	0.07565 (8)	0.0589 (5)	
O4	−0.0493 (2)	0.31104 (7)	0.07088 (6)	0.0382 (4)	
N2	0.1841 (2)	0.42193 (8)	0.08447 (7)	0.0317 (4)	
C13	0.8158 (3)	0.57440 (8)	0.15773 (7)	0.0256 (4)	
C14	0.6297 (3)	0.52971 (9)	0.11060 (8)	0.0292 (5)	
C15	0.5506 (3)	0.46122 (9)	0.15209 (8)	0.0334 (5)	
C16	0.4109 (3)	0.40679 (9)	0.10178 (8)	0.0324 (5)	
C17	0.0415 (3)	0.37297 (9)	0.03501 (8)	0.0341 (5)	
H1	0.03410	0.18950	0.16150	0.0290*	
H1A	0.290 (3)	0.1313 (10)	0.2847 (8)	0.027 (4)*	
H1B	0.038 (4)	0.1508 (10)	0.2824 (10)	0.046 (5)*	
H2A	0.52230	0.14590	0.17460	0.0360*	
H2B	0.42740	0.23010	0.16150	0.0360*	
H3A	0.24910	0.19800	0.04500	0.0400*	
H3B	0.52180	0.17420	0.04970	0.0400*	
H4A	0.27430	0.07640	−0.00350	0.0450*	
H4B	0.42380	0.04790	0.06940	0.0450*	
H5A	0.02890	0.00880	0.07180	0.0430*	
H5B	−0.06280	0.09340	0.05960	0.0430*	
H6A	−0.06090	0.06330	0.18450	0.0350*	
H6B	0.21240	0.04000	0.18840	0.0350*	
H7	0.39910	0.25640	0.27880	0.0320*	
H8A	0.12210	0.21370	0.39470	0.0410*	
H8B	0.39970	0.19870	0.39350	0.0410*	
H9A	0.33190	0.30530	0.46690	0.0500*	
H9B	0.47760	0.32860	0.40000	0.0500*	
H10A	0.26100	0.39360	0.28970	0.0670*	
H10B	−0.01650	0.40940	0.28940	0.0670*	
H11A	0.17100	0.41610	0.41020	0.0550*	
H11B	−0.02120	0.34990	0.40380	0.0550*	
H12A	−0.09990	0.27950	0.27980	0.0480*	
H12B	0.05430	0.30280	0.21490	0.0480*	
H2N	0.124 (3)	0.4572 (10)	0.1056 (9)	0.034 (5)*	
H4O	−0.195 (4)	0.3219 (13)	0.0796 (11)	0.068 (7)*	
H14A	0.69610	0.51300	0.06550	0.0350*	
H14B	0.49160	0.56220	0.09640	0.0350*	
H15A	0.69130	0.43540	0.17620	0.0400*	
H15B	0.45190	0.47800	0.19050	0.0400*	
H17A	−0.09140	0.40220	0.01050	0.0410*	
H17B	0.13900	0.35450	−0.00300	0.0410*	

### Atomic displacement parameters (Å^2^)

**Table:**

	*U*^11^	*U*^22^	*U*^33^	*U*^12^	*U*^13^	*U*^23^
N1	0.0259 (7)	0.0221 (7)	0.0210 (6)	0.0028 (6)	0.0002 (5)	0.0016 (5)
C1	0.0289 (8)	0.0222 (7)	0.0202 (7)	0.0021 (7)	−0.0007 (6)	−0.0006 (5)
C2	0.0358 (9)	0.0276 (8)	0.0276 (7)	−0.0042 (7)	0.0051 (6)	−0.0013 (6)
C3	0.0427 (10)	0.0324 (9)	0.0273 (8)	−0.0034 (8)	0.0104 (7)	−0.0013 (6)
C4	0.0526 (11)	0.0335 (9)	0.0278 (8)	0.0002 (8)	0.0096 (7)	−0.0069 (7)
C5	0.0478 (11)	0.0306 (9)	0.0293 (8)	−0.0065 (8)	0.0019 (7)	−0.0077 (6)
C6	0.0362 (9)	0.0245 (8)	0.0277 (7)	−0.0039 (7)	0.0021 (6)	−0.0008 (6)
C7	0.0314 (9)	0.0245 (8)	0.0248 (7)	−0.0036 (7)	0.0041 (6)	−0.0039 (6)
C8	0.0418 (10)	0.0342 (9)	0.0254 (7)	0.0073 (8)	−0.0033 (7)	−0.0040 (6)
C9	0.0476 (11)	0.0452 (10)	0.0319 (8)	−0.0037 (9)	−0.0003 (7)	−0.0125 (8)
C10	0.0968 (18)	0.0235 (9)	0.0439 (10)	0.0069 (10)	−0.0043 (10)	−0.0039 (7)
C11	0.0669 (14)	0.0300 (9)	0.0411 (9)	0.0003 (9)	0.0038 (9)	−0.0121 (7)
C12	0.0623 (12)	0.0257 (9)	0.0305 (8)	0.0080 (9)	−0.0069 (8)	0.0000 (6)
O1	0.0304 (6)	0.0338 (6)	0.0409 (6)	−0.0011 (5)	0.0038 (5)	−0.0132 (5)
O2	0.0265 (6)	0.0311 (6)	0.0485 (6)	0.0006 (5)	−0.0026 (5)	−0.0113 (5)
O3	0.0329 (7)	0.0372 (8)	0.1074 (12)	−0.0028 (6)	0.0107 (7)	−0.0271 (7)
O4	0.0313 (7)	0.0291 (6)	0.0540 (7)	−0.0021 (6)	0.0033 (6)	−0.0010 (5)
N2	0.0303 (8)	0.0277 (7)	0.0366 (7)	−0.0019 (6)	0.0011 (6)	−0.0095 (6)
C13	0.0273 (8)	0.0243 (8)	0.0251 (7)	−0.0030 (7)	0.0017 (6)	0.0007 (6)
C14	0.0288 (9)	0.0250 (8)	0.0325 (8)	−0.0037 (7)	−0.0040 (6)	0.0005 (6)
C15	0.0316 (9)	0.0309 (9)	0.0368 (8)	−0.0074 (8)	−0.0020 (7)	0.0026 (7)
C16	0.0300 (9)	0.0236 (8)	0.0441 (9)	−0.0056 (7)	0.0060 (7)	−0.0008 (7)
C17	0.0357 (9)	0.0335 (9)	0.0330 (8)	−0.0047 (8)	0.0023 (7)	−0.0056 (7)

### Geometric parameters (Å, °)

**Table:**

O1—C13	1.2505 (18)	C3—H3A	0.9900
O2—C13	1.259 (2)	C4—H4B	0.9900
O3—C16	1.232 (2)	C4—H4A	0.9900
O4—C17	1.408 (2)	C5—H5A	0.9900
O4—H4O	0.88 (2)	C5—H5B	0.9900
N1—C1	1.5068 (17)	C6—H6B	0.9900
N1—C7	1.5030 (19)	C6—H6A	0.9900
N1—H1A	0.892 (17)	C7—H7	1.0000
N1—H1B	0.95 (2)	C8—H8B	0.9900
N2—C16	1.326 (2)	C8—H8A	0.9900
N2—C17	1.451 (2)	C9—H9B	0.9900
N2—H2N	0.829 (17)	C9—H9A	0.9900
C1—C6	1.524 (2)	C10—H10B	0.9900
C1—C2	1.519 (2)	C10—H10A	0.9900
C2—C3	1.529 (2)	C11—H11B	0.9900
C3—C4	1.526 (2)	C11—H11A	0.9900
C4—C5	1.524 (2)	C12—H12B	0.9900
C5—C6	1.528 (2)	C12—H12A	0.9900
C7—C12	1.515 (2)	C13—C14	1.526 (2)
C7—C8	1.524 (2)	C14—C15	1.529 (2)
C8—C9	1.520 (2)	C15—C16	1.512 (2)
C9—C11	1.516 (3)	C14—H14A	0.9900
C10—C12	1.533 (2)	C14—H14B	0.9900
C10—C11	1.522 (2)	C15—H15A	0.9900
C1—H1	1.0000	C15—H15B	0.9900
C2—H2A	0.9900	C17—H17A	0.9900
C2—H2B	0.9900	C17—H17B	0.9900
C3—H3B	0.9900		
			
C17—O4—H4O	107.9 (15)	C5—C6—H6A	110.00
C1—N1—C7	117.20 (11)	N1—C7—H7	109.00
C7—N1—H1A	108.0 (11)	C8—C7—H7	109.00
C1—N1—H1A	109.8 (10)	C12—C7—H7	109.00
C1—N1—H1B	109.6 (11)	C7—C8—H8B	109.00
C7—N1—H1B	105.5 (11)	C9—C8—H8A	109.00
H1A—N1—H1B	106.2 (16)	C7—C8—H8A	109.00
C16—N2—C17	120.06 (14)	C9—C8—H8B	109.00
C16—N2—H2N	118.4 (12)	H8A—C8—H8B	108.00
C17—N2—H2N	121.3 (12)	C11—C9—H9B	109.00
N1—C1—C2	111.81 (13)	H9A—C9—H9B	108.00
C2—C1—C6	111.55 (13)	C8—C9—H9A	110.00
N1—C1—C6	107.59 (11)	C8—C9—H9B	110.00
C1—C2—C3	110.53 (13)	C11—C9—H9A	109.00
C2—C3—C4	111.38 (13)	C11—C10—H10B	109.00
C3—C4—C5	110.84 (13)	C12—C10—H10A	109.00
C4—C5—C6	110.95 (13)	C12—C10—H10B	109.00
C1—C6—C5	110.40 (12)	C11—C10—H10A	109.00
N1—C7—C8	107.79 (11)	H10A—C10—H10B	108.00
N1—C7—C12	110.86 (13)	C9—C11—H11B	110.00
C8—C7—C12	111.92 (13)	H11A—C11—H11B	108.00
C7—C8—C9	110.83 (13)	C10—C11—H11A	110.00
C8—C9—C11	110.60 (14)	C9—C11—H11A	110.00
C11—C10—C12	111.20 (14)	C10—C11—H11B	110.00
C9—C11—C10	110.29 (16)	C10—C12—H12B	110.00
C7—C12—C10	110.15 (16)	H12A—C12—H12B	108.00
N1—C1—H1	109.00	C7—C12—H12B	110.00
C6—C1—H1	109.00	C10—C12—H12A	110.00
C2—C1—H1	109.00	C7—C12—H12A	110.00
C3—C2—H2B	110.00	O1—C13—O2	123.51 (14)
C3—C2—H2A	110.00	O1—C13—C14	118.94 (15)
H2A—C2—H2B	108.00	O2—C13—C14	117.55 (13)
C1—C2—H2B	110.00	C13—C14—C15	110.60 (12)
C1—C2—H2A	110.00	C14—C15—C16	111.49 (12)
C2—C3—H3B	109.00	O3—C16—N2	121.23 (15)
H3A—C3—H3B	108.00	O3—C16—C15	121.41 (15)
C4—C3—H3B	109.00	N2—C16—C15	117.30 (14)
C2—C3—H3A	109.00	O4—C17—N2	112.52 (12)
C4—C3—H3A	109.00	C13—C14—H14A	110.00
C5—C4—H4B	109.00	C13—C14—H14B	110.00
H4A—C4—H4B	108.00	C15—C14—H14A	110.00
C5—C4—H4A	109.00	C15—C14—H14B	110.00
C3—C4—H4A	109.00	H14A—C14—H14B	108.00
C3—C4—H4B	109.00	C14—C15—H15A	109.00
C4—C5—H5B	109.00	C14—C15—H15B	109.00
H5A—C5—H5B	108.00	C16—C15—H15A	109.00
C4—C5—H5A	109.00	C16—C15—H15B	109.00
C6—C5—H5B	109.00	H15A—C15—H15B	108.00
C6—C5—H5A	109.00	O4—C17—H17A	109.00
C1—C6—H6B	110.00	O4—C17—H17B	109.00
H6A—C6—H6B	108.00	N2—C17—H17A	109.00
C5—C6—H6B	110.00	N2—C17—H17B	109.00
C1—C6—H6A	110.00	H17A—C17—H17B	108.00
			
C7—N1—C1—C2	59.28 (18)	C4—C5—C6—C1	56.57 (17)
C7—N1—C1—C6	−177.90 (14)	N1—C7—C8—C9	−177.94 (14)
C1—N1—C7—C8	−176.66 (14)	C8—C7—C12—C10	54.9 (2)
C1—N1—C7—C12	60.53 (19)	C12—C7—C8—C9	−55.78 (19)
C16—N2—C17—O4	84.82 (17)	N1—C7—C12—C10	175.30 (14)
C17—N2—C16—O3	1.2 (2)	C7—C8—C9—C11	56.73 (18)
C17—N2—C16—C15	178.48 (13)	C8—C9—C11—C10	−57.9 (2)
N1—C1—C2—C3	176.74 (12)	C12—C10—C11—C9	57.7 (3)
C2—C1—C6—C5	−56.83 (17)	C11—C10—C12—C7	−55.9 (2)
C6—C1—C2—C3	56.21 (16)	O1—C13—C14—C15	−96.94 (16)
N1—C1—C6—C5	−179.81 (14)	O2—C13—C14—C15	82.16 (17)
C1—C2—C3—C4	−55.54 (17)	C13—C14—C15—C16	−166.86 (13)
C2—C3—C4—C5	55.81 (17)	C14—C15—C16—O3	95.92 (18)
C3—C4—C5—C6	−56.27 (17)	C14—C15—C16—N2	−81.33 (18)

### Hydrogen-bond geometry (Å, °)

**Table:**

*D*—H···*A*	*D*—H	H···*A*	*D*···*A*	*D*—H···*A*
N1—H1A···O1^i^	0.892 (17)	2.597 (17)	3.285 (2)	134.7 (14)
N1—H1A···O2^i^	0.892 (17)	1.975 (17)	2.8546 (18)	168.7 (15)
N1—H1B···O1^ii^	0.95 (2)	1.80 (2)	2.740 (2)	174.3 (17)
N2—H2N···O2^iii^	0.829 (17)	2.069 (17)	2.8914 (18)	171.1 (16)
O4—H4O···O3^iii^	0.88 (2)	1.78 (2)	2.6423 (16)	166.4 (19)

Symmetry codes: (i) −*x*+3/2, *y*−1/2, −*z*+1/2; (ii) −*x*+1/2, *y*−1/2, −*z*+1/2; (iii) *x*−1, *y*, *z*.
